# Exercise stress test unmasking a Brugada pattern in a survivor of cardiac arrest: a case report

**DOI:** 10.1093/ehjcr/ytaf673

**Published:** 2025-12-23

**Authors:** Margarida Castro, Luísa Pinheiro, Sílvia Ribeiro, Victor Manuel Sanfins, António Lourenço

**Affiliations:** Cardiology Department, Unidade Local de Saúde do Alto Ave, Rua dos Cutileiros – Creixomil, 4835-044 Guimarães, Portugal; Cardiology Department, Unidade Local de Saúde do Alto Ave, Rua dos Cutileiros – Creixomil, 4835-044 Guimarães, Portugal; Arrhythmology Department, Unidade Local de Saúde do Alto Ave, Rua dos Cutileiros – Creixomil, 4835-044 Guimarães, Portugal; Arrhythmology Department, Unidade Local de Saúde do Alto Ave, Rua dos Cutileiros – Creixomil, 4835-044 Guimarães, Portugal; Cardiology Department, Unidade Local de Saúde do Alto Ave, Rua dos Cutileiros – Creixomil, 4835-044 Guimarães, Portugal

**Keywords:** Brugada syndrome, Exercise stress test, Sudden cardiac arrest, Type 1 Brugada pattern, Risk stratification, Case report

## Abstract

**Background:**

Brugada syndrome (BrS) is a hereditary arrhythmic disorder associated with an increased risk of ventricular arrhythmias (VA) and sudden cardiac death (SCD). Certain triggers may unmask the diagnostic high-risk BrS Type 1 electrocardiographic (ECG) pattern. However, conversion to a Type 1 pattern during peak exercise testing is uncommon. Despite the established benefits of physical activity, exercise in patients with inherited arrhythmia syndromes may provoke malignant arrhythmias. Current guidelines allow for some degree of sports participation in asymptomatic individuals with BrS, including mutation carriers and athletes with only an inducible ECG pattern.

**Case summary:**

We describe a case of a young man who survived a cardiac arrest during physical exertion. A treadmill stress test revealed a diagnostic Type 1 BrS ECG pattern at peak exercise. The resting ECG was non-diagnostic, and comprehensive evaluation excluded other causes of cardiac arrest.

**Discussion:**

In BrS, VA most commonly occur at rest, during sleep, or during exercise recovery, when vagal tone is predominant. This case demonstrates that BrS Type 1 ECG changes may also be elicited during peak sympathetic stimulation, such as intense exercise. This rare presentation suggests that exercise stress testing could play a role in the diagnostic and risk stratification process in selected patients with suspected BrS. Furthermore, it raises questions regarding the safety of physical activity in this population and whether current recommendations on sports participation warrant re-evaluation in light of such findings.

Learning points

- Exercise testing may unmask a Type 1 Brugada pattern in asymptomatic patients.
- Although not currently part of diagnostic criteria, exercise testing can reveal malignant phenotypes and support clinical decision-making.
- Type 1 Brugada ECG pattern during peak exercise suggests sympathetic or rate-related triggers and justify restricting vigorous physical activity.

## Introduction

Brugada syndrome (BrS) is an inherited channelopathy that typically affects patients with structurally normal hearts and increases the risk of ventricular arrhythmias (VA) and sudden cardiac death (SCD).^1^ Certain triggers, such as fever, increased vagal tone, and sodium channel blockers, can unmask a Type 1 Brugada ECG pattern.^2^ Data on the risk of exercise in BrS patients are limited, and conversion to a Type 1 pattern during exertion is uncommon.^3^

## Summary figure

**Figure:**
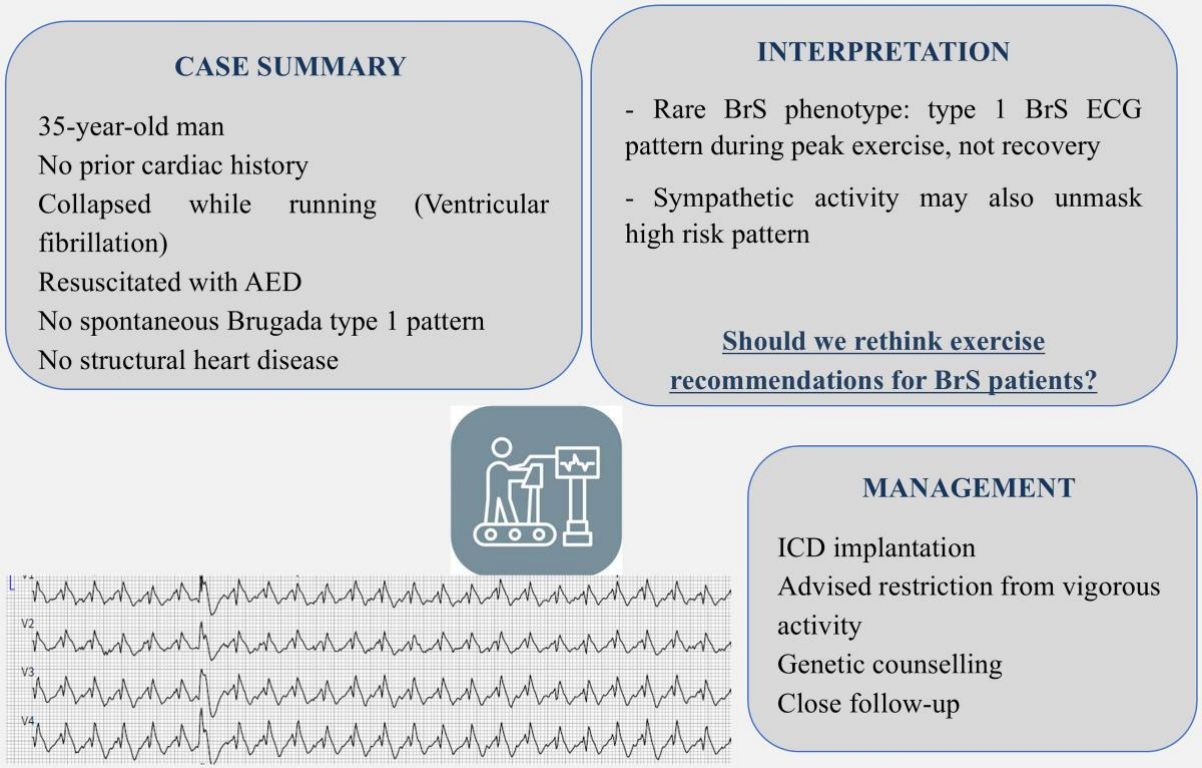


## Case presentation

We report the case of a 35-year-old previously asymptomatic man who survived an out-of-hospital cardiac arrest and displayed a Type 1 Brugada ECG pattern during an exercise stress test.

The patient collapsed while running and was witnessed by a nurse outside a fire station. Advanced life support was initiated; a single shock from an automated external defibrillator terminated ventricular fibrillation.

**Figure 1:**
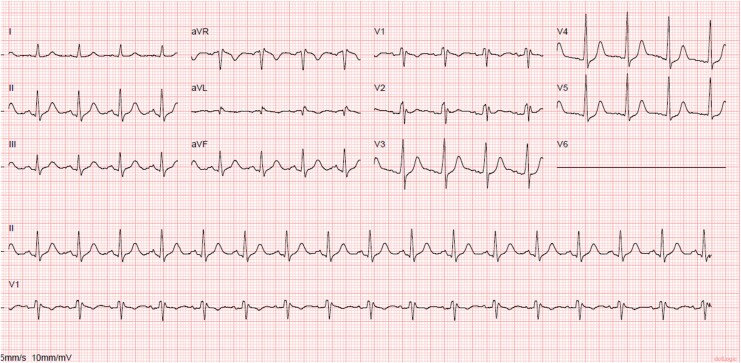
Admission electrocardiogram showing incomplete right branch block with normal QT interval.

**Figure 2:**
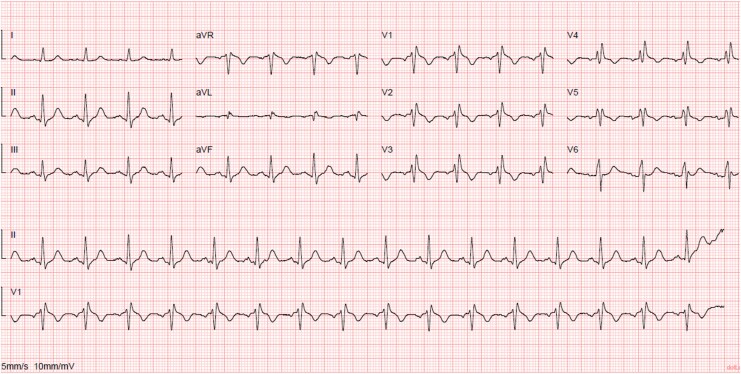
Modified electrocardiogram with high right precordial leads showing incomplete right bundle branch block with ST-segment elevation of 1 mm, with no record of Type 1 Brugada pattern (V1–V2 corresponds to V1–V2 on the second intercostal space, V3–V4 on the third intercostal space,and V5–V6 on the fourth intercostal space).

On arrival at the emergency department, he was afebrile, haemodynamically stable, and intubated. The admission ECG showed an incomplete right bundle branch block with a normal QT interval (*Figure 1*). A modified high right precordial lead (RPL) ECG demonstrated 1 mm ST-segment elevation but no Type 1 pattern (*Figure 2*). Transthoracic echocardiography and laboratory tests, including cardiac biomarkers and serum electrolytes, were unremarkable.

His medical history was notable for nodular Hodgkin lymphoma under oncological follow-up; there was no family history of SCD or cardiovascular disease. His intensive care stay was uncomplicated, with uneventful weaning from sedation and mechanical ventilation.

Coronary computed tomography angiography excluded coronary artery disease and anomalous coronary origin, and cardiac magnetic resonance imaging showed no structural abnormalities, including absence of oedema and fibrosis.

**Figure 3:**
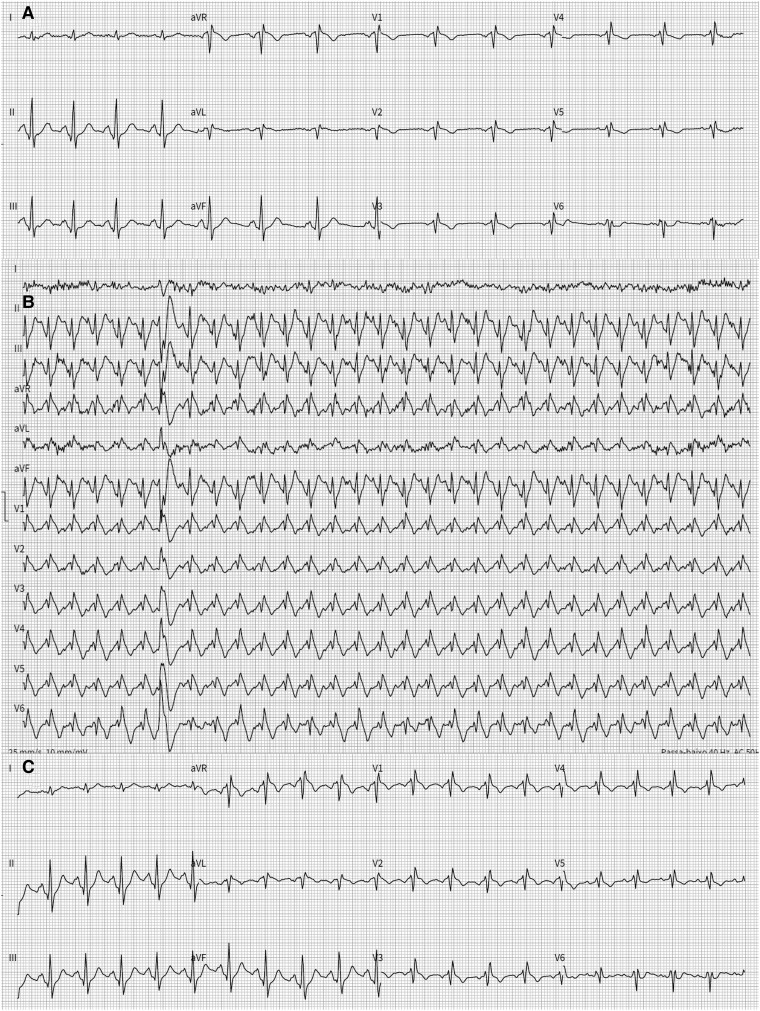
Electrocardiogram results on exercise stress test. (A) Baseline modified electrocardiogram with high right precordial lead. (B) Maximum ST-segment augmentation with transition to the Type 1 pattern (circle) occurring during peak exercise at a heart rate of 186 b.p.m. (C) Electrocardiograms representing termination of testing at 5 min into recovery.

To reproduce the circumstances of the arrest, a treadmill exercise stress test (high RPLs) was performed to exclude catecholaminergic polymorphic ventricular tachycardia. At peak exercise (heart rate 174–186 b.p.m.) intermittent electrical alternans with a Type 1 Brugada pattern was documented; ST-segment changes resolved gradually during recovery. The patient completed Stage 5 of a standard Bruce protocol without VAs (*Figure 3*).

An electrophysiology study was not pursued, given its limited role in BrS and the fact that a negative result would not have changed the indication for implantable cardioverter-defibrillator (ICD), after careful decision involving the patient.

An extended genetic panel for primary electrical diseases, including SCN5A and other channelopathy-associated genes, was performed and yielded negative results, and a subcutaneous ICD was subsequently implanted.

At 12-month follow-up, he remains asymptomatic, free of arrhythmic events, and has been advised to avoid moderate- to high-intensity exercise. The patient’s family underwent ajmaline provocation testing, and one of the siblings developed a Type 1 Brugada pattern.

## Discussion

Brugada syndrome is a rare genetic channelopathy that accounts for up to 20% of sudden deaths in young adults with structurally normal hearts. More than 80% of VAs occur during periods of high vagal tone, typically at night or during rest.^2^ Reports of exercise-related symptoms are scarce and usually show ST-segment augmentation in the recovery phase, reinforcing the role of parasympathetic activity in BrS arrhythmogenesis.^2,4–6^

Exercise stress testing is considered safe in BrS and may aid both diagnosis and risk stratification. Adverse prognostic markers include an early-recovery ST-segment rise >0.5 mV in V1–V3, late-recovery J-point elevation >2 mm in lead aVR, < 40% heart-rate fall from peak in late recovery, and ventricular ectopy in early recovery.^7^ Recording high right precordial leads at rest improves detection of the Type 1 pattern by approximately 32%.^8^

Considering that our patient presented with cardiorespiratory arrest, it was decided not to perform a pharmacological provocation test with Ajmaline, and the diagnosis was assumed based on the exercise test, although the stress test is not included in the guidelines as a diagnostic tool. Masrur *et al*. described the effects of exercise stress testing on BrS patients. In a series of 166 BrS patients, Masrur *et al*. reported ST-segment augmentation in 57%, but only two cases occurred at peak exercise.^3^ Our case therefore represents a rare phenotype in which sympathetic activation or tachycardia alone unmasks the Type 1 pattern; hyperthermia may also contribute.^9^

A distinct SCN5A mutation that enhances slow inactivation and delays sodium channel recovery has been linked to ST-segment augmentation at high heart rates.^10,11^ Our patient was SCN5A-negative, suggesting additional genetic substrates remain to be identified; such phenotypes may also respond poorly to isoprenaline in electrical storm.

The possibility of dual pathology, namely, a Brugada phenotype coexisting with idiopathic ventricular fibrillation (IVF), cannot be entirely excluded, particularly given that IVF remains a diagnosis of exclusion. Historically, many cases labelled as IVF were later recognized to reflect underdiagnosed primary arrhythmia syndromes, largely due to limited diagnostic resources and the absence of genetic testing. With advances in electrocardiographic, imaging, and genetic evaluation, the incidence of true IVF has progressively declined, as conditions such as BrS, CPVT, long-QT and short-QT syndromes, and early repolarization syndrome have been identified as distinct entities rather than subsets of IVF. Furthermore, genetic discoveries, including variants in DPP6, CALM1, RyR2, and IRX3, have broadened the understanding of inherited arrhythmogenic disorders once classified as IVF.^12^

Risk stratification with an electrophysiological study in BrS remains controversial, and therefore, it was not pursued following shared decision-making with the patient. The subsequent finding of a positive ajmaline provocation test in a first-degree relative further supports the diagnosis of BrS in this case.

Data on exercise risk in BrS remain limited. Current expert consensus (Class IIb) allows asymptomatic individuals and athletes and genotype-positive relatives to engage in sports that do not raise core temperature, provided an automated external defibrillator is available.^13^ Symptomatic patients with an ICD may resume activity, including leisure or competitive sports, after three uneventful months through shared decision-making.^13^

However, our observation that vigorous exercise elicited a malignant ECG phenotype supports restricting high-intensity exercise in selected BrS patients pending further evidence.

This case adds to the unusual findings that a Type 1 Brugada pattern can emerge at peak exercise and highlights the potential role of stress testing in risk stratification. Larger studies are needed to define the prognostic value of exercise-induced ECG changes and to guide exercise recommendations in BrS.

## Lead author biography


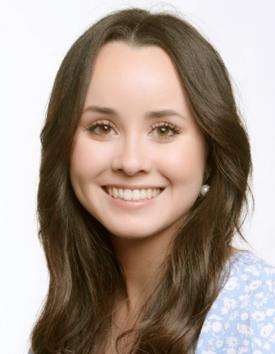


Margarida Castro is currently a last year cardiology resident at the Unidade Local de Saúde do Alto Ave, Portugal. She graduated from the Faculty of Medicine of University of Coimbra. She is interested and doing research on acute cardiac intensive care, heart failure, and arrhythmias.

## Data Availability

All data are incorporated into the article and its online supplementary material.
